# The methyltransferase HEN1 is required in *Nematostella vectensis* for microRNA and piRNA stability as well as larval metamorphosis

**DOI:** 10.1371/journal.pgen.1007590

**Published:** 2018-08-17

**Authors:** Vengamanaidu Modepalli, Arie Fridrich, Maayan Agron, Yehu Moran

**Affiliations:** Department of Ecology, Evolution and Behavior, Alexander Silberman Institute of Life Sciences, Faculty of Science, The Hebrew University of Jerusalem, Jerusalem, Israel; University of Cambridge, UNITED KINGDOM

## Abstract

Small non-coding RNAs (sRNAs) such as microRNAs (miRNAs), small interfering RNAs (siRNAs) and piwi-interacting RNAs (piRNAs) regulate the levels of endogenous, viral and transposable element RNA in plants (excluding piRNAs) and animals. These pathways are explored mainly in bilaterian animals, such as vertebrates, arthropods and nematodes, where siRNAs and piRNAs, but not miRNAs bind their targets with a perfect match and mediate the cleavage of the target RNA. Methylation of the 3′ ends of piRNAs and siRNAs by the methyltransferase HEN1 protects these sRNAs from degradation. There is a noticeable selection in bilaterian animals against miRNA-mRNA perfect matching, as it leads to the degradation of miRNAs. Cnidarians (sea anemones, corals, hydroids and jellyfish), are separated from bilaterians by more than 600 million years. As opposed to bilaterians, cnidarian miRNAs frequently bind their targets with a nearly perfect match. Knowing that an ortholog of HEN1 is widely expressed in the sea anemone *Nematostella vectensis*, we tested in this work whether it mediates the stabilization of its sRNAs. We show that the knockdown of HEN1 in *Nematostella* results in a developmental arrest. Small RNA sequencing revealed that the levels of both miRNAs and piRNAs drop dramatically in the morphant animals. Moreover, knockdown experiments of *Nematostella* Dicer1 and PIWI2, homologs of major bilaterian biogenesis components of miRNAs and piRNAs, respectively, resulted in developmental arrest similar to HEN1 morphants. Our findings suggest that HEN1 mediated methylation of sRNAs reflects the ancestral state, where miRNAs were also methylated. Thus, we provide the first evidence of a methylation mechanism that stabilizes miRNAs in animals, and highlight the importance of post-transcriptional regulation in non-bilaterian animals.

## Author summary

Plants and animals use small RNAs to regulate gene expression, virus silencing and genomic integrity. These functions depend on specific binding of small RNAs to longer RNA targets. The methyltransferase HEN1 plays a crucial role in stabilizing small RNAs upon their binding to perfectly-matching targets. Lack of methylation in case of a perfect match will lead to small RNA degradation. In general, methylation of microRNAs, a class of small RNAs, is part of their biogenesis in plants, but not in bilaterian animals such as vertebrates, worms and insects, where perfectly-matching microRNA targets are rare. In contrast, in Cnidaria (sea anemones, corals and jellyfish), the sister group to Bilateria, microRNAs frequently bind their targets with a nearly perfect match. In this study, we show that in the cnidarian *Nematostella vectensis* methylation of microRNAs and other small RNAs is consistent and frequent throughout development and that knockdown of the cnidarian HEN1 results in a developmental arrest. Small RNA sequencing of the treated animals, reveals that small RNAs are depleted and shortened. Therefore, our findings suggest that HEN1-mediated methylation of small RNAs was present in the last common ancestor of Cnidaria and Bilateria 600 million years ago and was responsible for microRNA stabilization.

## Introduction

**Fig 1:**
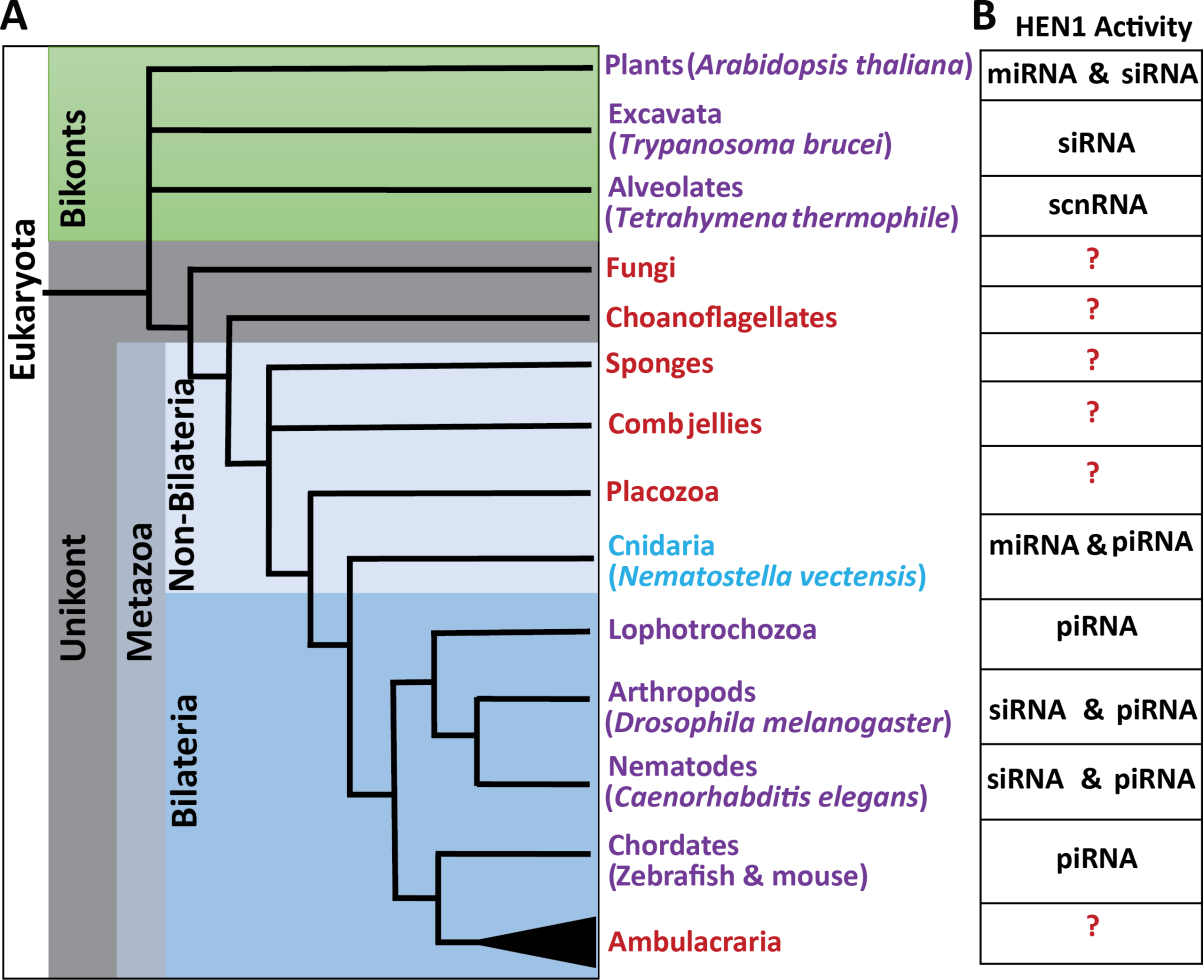
Schematic phylogenetic tree of Eukaryota at the phylum level. (A) Phyla where HEN1 protein was functionally studied appear in purple. Phyla where HEN1 homologs are found in the genome but have not been functionally studied yet appears in red. The sponges (Porifera) and comb jellies (Ctenophora) were illustrated as polytomy due to the current uncertainty regarding their relative phylogenetic positions. (B) A table indicating the type of sRNAs methylated by HEN1 in respective species is provided.

MicroRNAs (miRNAs) are small non-coding RNAs of ~22 nucleotides that bind to messenger RNAs (mRNAs) and mediate their cleavage, destabilization and translation inhibition in plants and animals [1–3]. Cleavage of mRNAs is executed directly by Argonautes (AGOs), the protein carriers of small RNAs (sRNAs) [4]. Translational inhibition and destabilization of mRNAs is mediated by AGO partner proteins such as SUO in plants [5], and GW182 in animals [4, 6]. In bilaterian animals, which compose the vast majority of extant animals, the level of complementarity between miRNAs and their targets dictates the fate of the target mRNA: only a nearly perfect match promotes the target’s cleavage. However, most of the bilaterian miRNAs bind to their targets with a short “seed” match of nucleotide positions 2–8 at the 5′ end of the miRNA. This type of interaction results in translational inhibition and destabilization of the mRNAs [4]. On the contrary, plant miRNAs require a full match to perform both, cleavage and translational inhibition and destabilization of the target, and a “seed” match alone would not result in any type of target downregulation [7]. Other types of sRNAs, such as small interfering RNA (siRNAs) and Piwi-interacting RNAs (piRNA) that target viruses and transposable elements, respectively, usually bind their RNA targets with a perfect match and cleave them [8, 9], although some exceptions were reported [10, 11]. Plants do not have piRNAs, and their transposable elements are silenced by siRNAs [12, 13]. Notably, full complementarity forces the 3′ end of sRNA strand out of the AGO PAZ domain, exposing them to exonucleases [14, 15]. The 2′-O-methylation at the 3′ end of these fully matching sRNA protects them from degradation. The methyltransferase HUA ENHANCER1 (HEN1) was shown to mediate this modification [16–18]. In bilaterians, only piRNAs and siRNAs are methylated while miRNAs are not methylated [18–20] (Fig 1) and a full miRNA-mRNA match in bilaterians results in the degradation of the unmethylated miRNA [21]. Studies of sRNA in Bilateria and higher plants have provided important mechanistic insights. However, little focus has been addressed to such mechanisms in non-bilaterian animals (Fig 1), where such findings would expand our knowledge about the function of this system and will deepen the understanding of the evolution of sRNA pathways.

Cnidaria (sea anemones, corals, hydroids and jellyfish) is the sister group of Bilateria, separated by more than 600 million years. Unlike their bilaterian counterparts, cnidarian miRNAs frequently bind to their targets with a high complementarity and mediate target cleavage [22]. This type of interaction between miRNAs and their targets might be a remnant of an ancestral mode of action, before the seed based recognition evolved [3]. Interestingly it was shown that in cnidarians not only piRNAs, but also miRNAs are methylated, most likely to stabilize the miRNAs during interaction with their target through a high complementarity matching [22, 23]. We hypothesized that the cnidarian ortholog of HEN1 might be responsible for this modification, and tested its function on the stability of sRNAs and development of the sea anemone *Nematostella vectensis*, a cnidarian lab model with readily available genetic manipulation tools [24, 25]. In addition, the small RNA pathway components were well defined in *Nematostella* among non-bilaterian metazoans. We know from our previous studies [22, 26], *Nematostella* possess known miRNA pathway components such as two homologs of the RNAse III Dicer [27], responsible for miRNA processing and two AGO proteins [26]. Additionally, two homologs of PIWI were identified, a clade of AGO proteins specialized in piRNA processing and carrying out their function [8, 26].

We show that HEN1-mediated methylation of miRNAs is prevalent throughout development and that HEN1 depletion results in a developmental arrest and a drop in the abundance and length of miRNAs and piRNAs. We further demonstrate developmental arrest by Dicer and PIWI knockdowns, supporting the importance of these sRNAs for the normal development of *Nematostella*. These results shed light on the evolution of sRNA stability and function and suggest that miRNAs were methylated before the bilaterian seed based mechanism evolved.

## Results

### miRNAs are frequently methylated throughout development of *Nematostella*

In our previous study [28] we observed that similarly to plants and bilaterians, *Nematostella* miRNAs exhibit heterogeneity in length [21, 29]. In *Drosophila*, this heterogeneity is dynamic and the length of a few miRNAs is increased with age. In ageing flies, these longer isoforms are loaded into the siRNA carrier AGO2, instead of the miRNA carrier AGO1, and exhibit 2′-O-methylation at their 3′ ends [30]. Based on these finding in *Drosophila*, we decided to test whether miRNA methylation is a developmentally regulated process in *Nematostella*.

**Fig 2:**
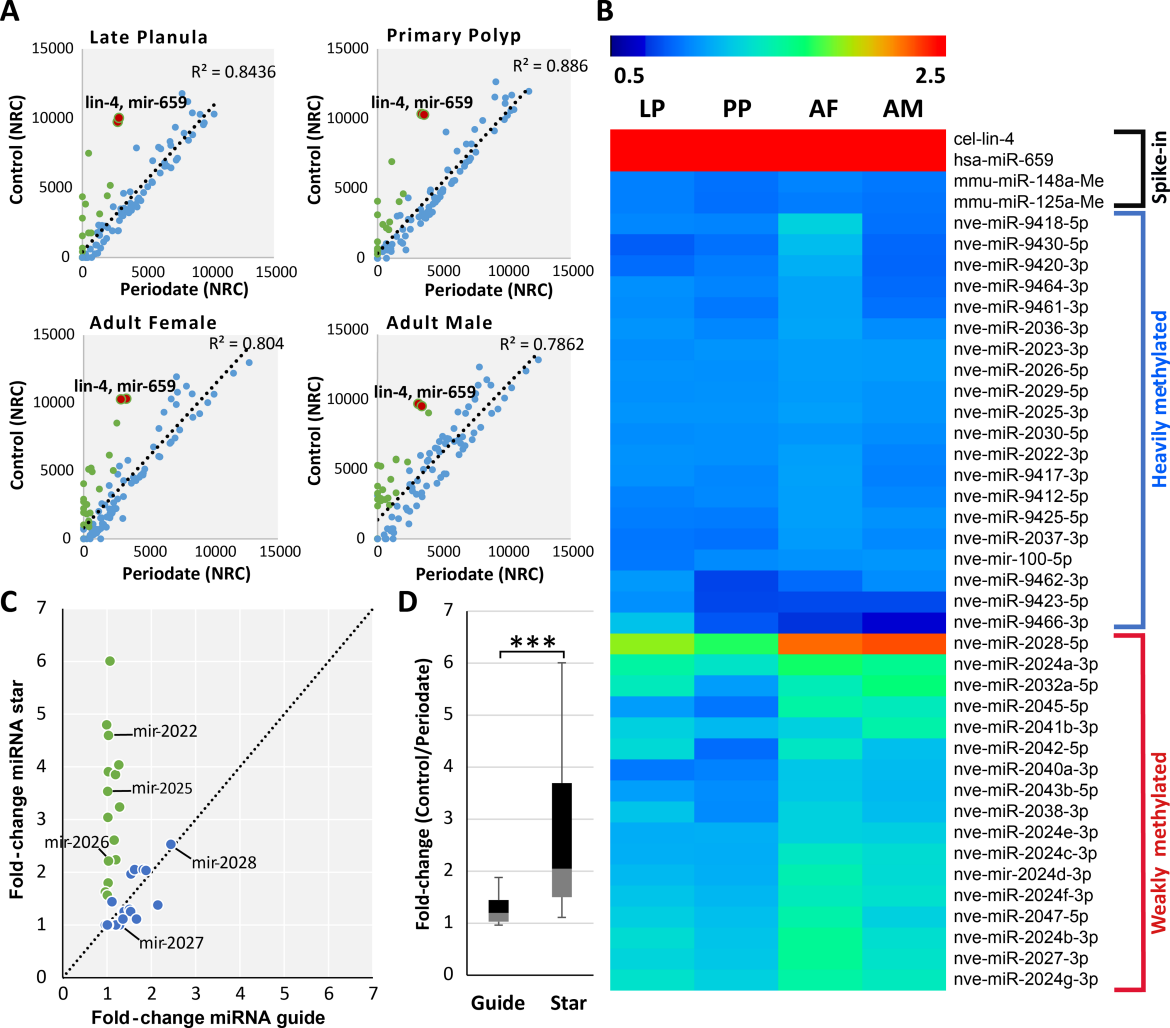
In *Nematostella vectensis* the miRNAs are frequently methylated and methylation frequencies are stable during development. (A) Scatter plot presenting the change in normalized read counts of individual miRNAs in control and periodate treated libraries. In blue, miRNAs whose levels changed less than two-fold. In green, miRNAs whose levels dropped two-fold or more. The majority of the miRNAs showed little to no change after periodate treatment. The non-methylated spike-ins are indicated as red dots in the scatterplot to demonstrate the efficiency of the periodate treatment. The axes are scaled to normalized read counts (NRC). The data represents the mean of three independent biological replicates. (B) Heatmap displaying log_2_-fold change of miRNA read counts between periodate treated and untreated samples in late planula (LP), primary polyp [9], adult female and male (AF and AM). The data is divided into two major clusters of heavily and weakly methylated miRNAs based on the fold change. To reduce noise, lowly expressed miRNAs (less than 50 read counts for a individual miRNA) were excluded from this analysis. (C) Depletion of miRNA* upon periodate treatment. The scatterplot represents the fold change in read counts of guide and star sequences of individual miRNAs before and after periodate treatment. Ratio of fold changes equal or larger than 1.5 are indicated in green. The miRNA* of miR-2022, miR-2025 and miR-2026 showed significantly higher fold-change compared to their guide sequences. Guides of moderately and weakly methylated miRNAs such as miR-2027 and miR-2028 showed a similar fold change to their stars. The results are presented in a box-plot in (D) showing the overall higher fold-change for star sequences compared to their guides. The box plot presenting the mean fold change for miRNA and miRNA* analyzed from planula larvae, primary polyps, adult male and female. P < 0.00001, Mann-Whitney test, miRNA *n* = 31.

We measured miRNA methylation levels in *Nematostella* at three developmental stages: planula larvae, primary polyps, and adults. We sequenced triplicate sRNAs libraries for each developmental stage. To evaluate the efficiency of the periodate treatment and to normalize the data, prior to sRNA library preparation four synthetic bilaterian miRNA were added as spike-ins to the total RNA, among them two were 2′-O-methylated at their 3′ end and two were non-methylated. To assess the miRNA methylation after sRNA size selection we subjected one half of each sample to periodate treatment [31] leaving the other half untreated. The ratio of non-methylated spike-ins between control to periodate treatment was used as an indication for the efficiency of periodate treatment. We observed a significant reduction (~ 1000 fold change) of those two non-methylated spike-ins in periodate treatment samples compared to untreated samples (S1A Fig). Our analysis revealed that all miRNAs were methylated in all developmental stages at least at some level, as noticeable by their comparison to the non-methylated spike-ins (Fig 2A and 2B; S2 Table). The log_2_-fold change between treated and untreated samples was calculated for each individual miRNA across developmental stages, based on the fold change we categorized the miRNAs into heavily methylated and weakly methylated clusters as presented in the heatmap (Fig 2B). Unlike *Drosophila* where miRNA methylation is age-associated [30], *Nematostella* miRNAs are methylated throughout development.

In the last step of miRNA biogenesis, a double-stranded duplex of sRNAs is loaded into an AGO, and strand selection occurs [32]. In this step, one strand is preferentially chosen to act as the guide miRNA, while the other, known as the miRNA-star (miRNA*) or passenger strand, is discarded. Unlike the plant HEN1, which bears double-strand RNA binding domains and methylates both strands of the duplex [33–35], in animals HEN1 was shown to methylate only a single strand of siRNAs and piRNAs [19, 36, 37]. Since we could not detect a double strand RNA binding domain in *Nematostella* HEN1 we expected the miRNA* not to be methylated. To test this hypothesis, we selected highly abundant miRNAs (n = 31), from different developmental stages and compared the methylation levels of miRNAs and miRNA*. Indeed, unlike guide miRNAs, the read counts of miRNA* tended to be much lower after periodate treatment (P < 0.0001, Mann-Whitney test) (Fig 2C and 2D) (S1B and S1C Fig). For example, the guide sequence of the miRNAs miR-2022, miR-2025 and miR-2026 have shown little to no fold-change between treated and untreated libraries. However, their star sequences showed a greater than two-fold change between the treatments (S1C Fig). This is in contrast to weakly methylated miRNAs like miR-2027 and miR-2028, where a similar drop in the read counts was observed for both their guide and star sequences (Fig 2C). Overall, we found that many miRNAs in *Nematostella* are methylated throughout development, and their guide, but not star sequences, are methylated.

### HEN1 depletion interferes with *Nematostella* development

**Fig 3:**
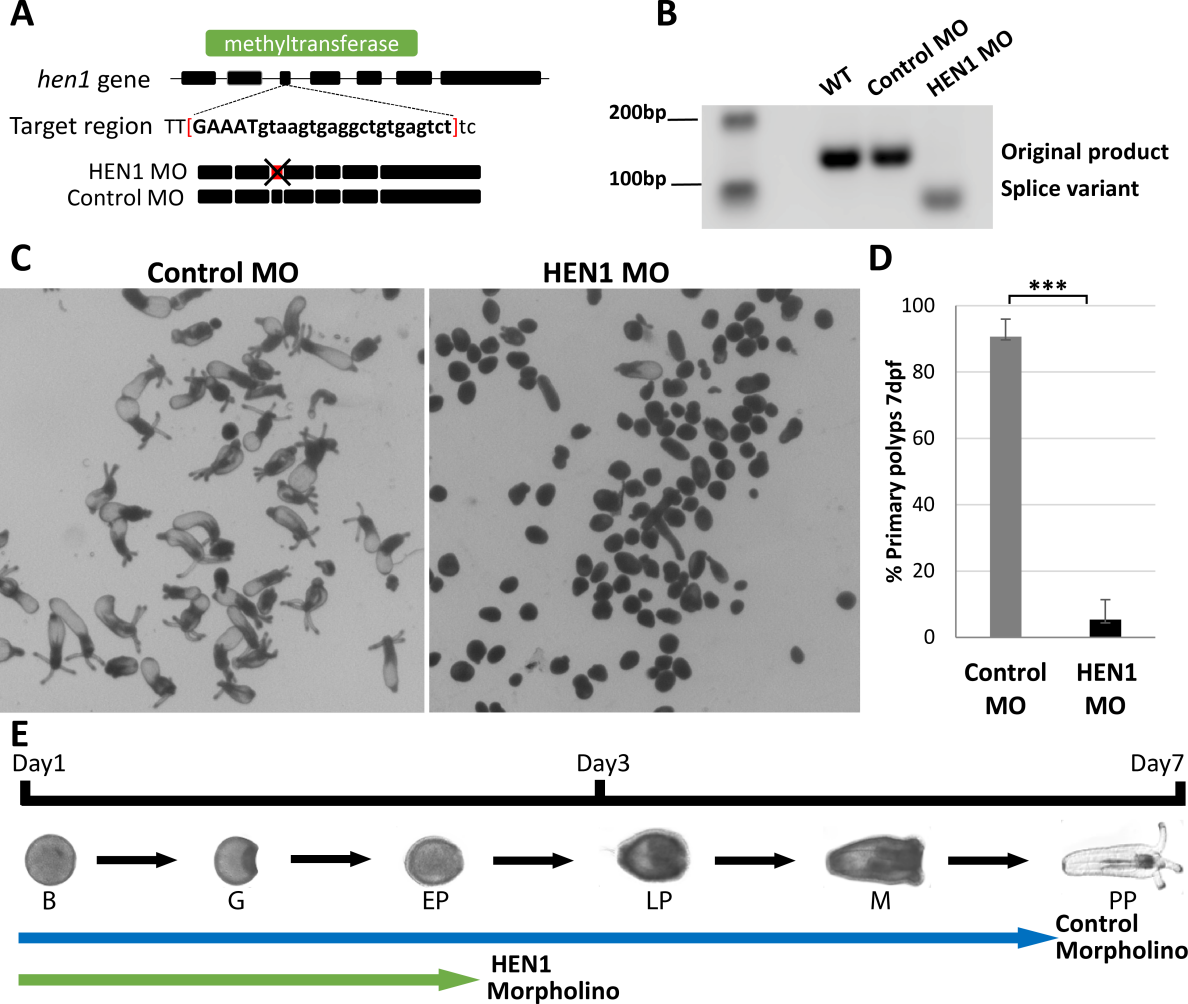
HEN1 is essential for *Nematostella* development. (A-B) Schematic diagram of MO targeting region on the *hen1* gene of *Nematostella*. The MO is designed to target *hen1* exon-intron junction located in methyltransferase domain (green). This MO impaired the splicing by deleting 3^rd^ exon of *hen1*. The splicing variation was validated by PCR. Due to deletion of 3^rd^ exon, the band in HEN1 MO-injected embryos shifted down. In contrast, the bands in control MO-injected embryos and wildtype presented the expected size. (C-D) Animals injected with control MO developed to primary polyps after 7 dpf. In contrast, animals injected with HEN1 MO stopped developing prior to metamorphosis (D) ~90% of HEN1 depleted animals did not reach primary polyp stage at 7 dpf, triplicates, n = 300, ***P < 0.001 (Student’s *t*-test). (E) The timeline of *Nematostella* development and the relative progress of control and HEN1 MO-injected animals. B = Blastula; G = Gastrula; EP = Early Planula; LP = Late Planula; M = Metamorphosis; PP = Primary Polyp.

In *Arabidopsis*, depletion of HEN1 reduces miRNA abundance and causes developmental abnormalities [38–40]. In bilaterian animals such as *C*. *elegans*, zebrafish, *Drosophila*, and mice, the depletion of HEN1 alters mostly spermatogenesis and ovulation and results in sterility [18, 19, 41–44]. These defects are related to the function of the sRNA populations that need to be methylated by HEN1. In non-bilaterian animals like *Nematostella*, role of HEN1 in stabilizing sRNAs is unknown. In a previous study [22] we observed that *Nematostella* HEN1 is expressed ubiquitously throughout the animal from early development, suggesting that unlike in most bilaterians, HEN1 in *Nematostella* is expressed in somatic cells. Thus, to investigate its functional role, we depleted HEN1 in embryos by injecting splice Morpholino Oligonucleotide (MO) targeting an exon-intron junction in the methyltransferase coding region of HEN1 (Fig 3A). Additionally, we also inhibited HEN1 translation by injecting translation blocking MO targeting the 5′ UTR of *hen1* mRNA. The *hen1* splice variant was confirmed by PCR amplification of a targeted region of RNA (Fig 3B). In parallel to these knockdown treatments, a control group of embryos were also injected with a standard control-MO with no target in the genome. The animals were examined until 10-days post fertilization (dpf). The control MO-injected embryos developed normally, undergone metamorphosis and progressed into primary polyps (Fig 3C). In contrast, the HEN1 MO-injected embryos showed developmental defects (Fig 3C) as by 7 dpf ~90% of these larvae stopped developing and remained at the planula stage (Fig 3D). This phenotype was highly reproducible by both HEN1 splice MO and translation blocking MO (S2 Fig). Developmental defects in HEN1-depleted animals suggest that this methyltransferase is essential for *Nematostella* development.

### HEN1 depletion reduces the stability of miRNAs in *Nematostella*

**Fig 4:**
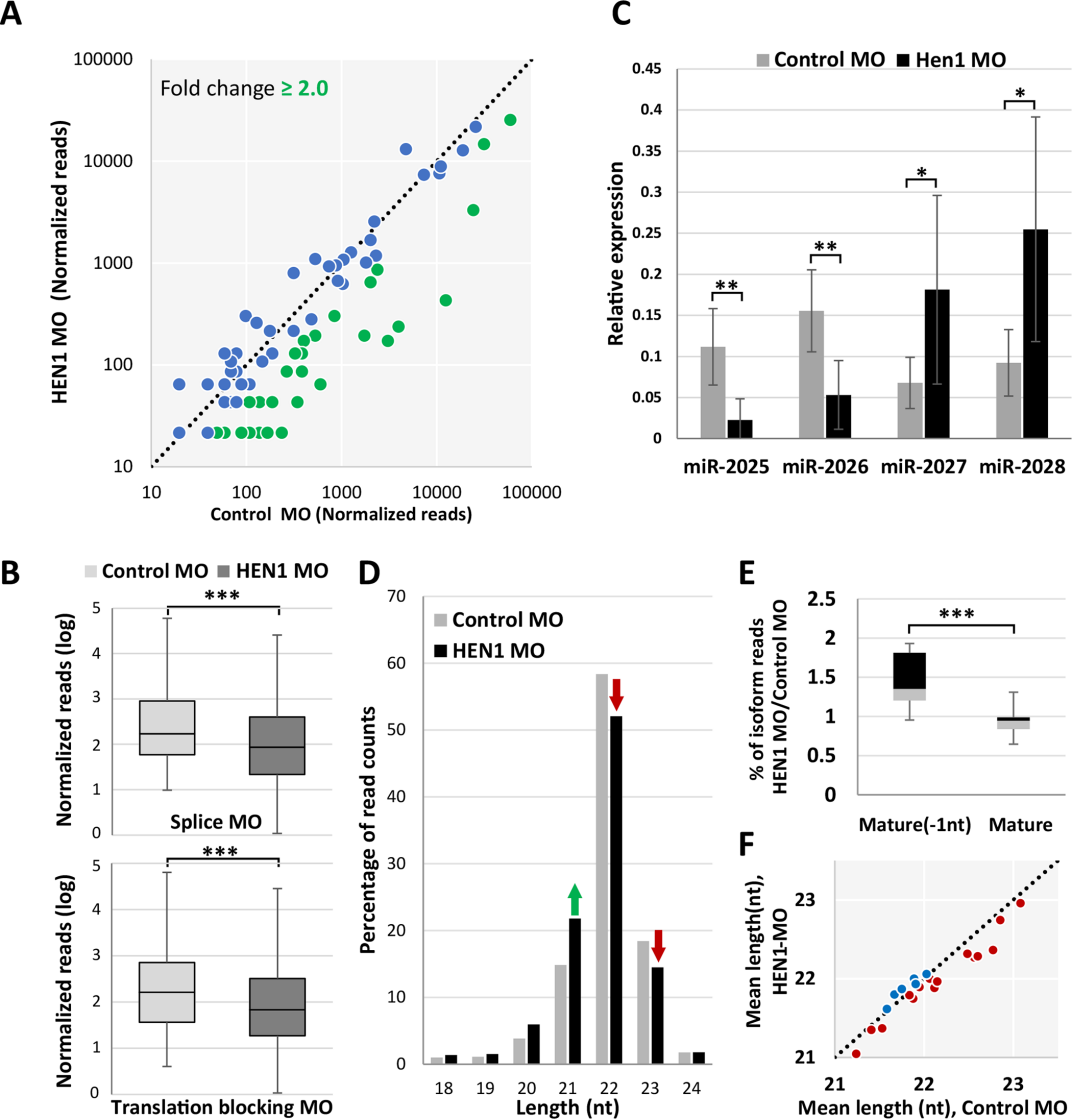
HEN1 is required for miRNA stability. (A) Change in miRNA read counts after HEN1 depletion is depicted by scatter plot. Each dot represents the read counts of an individual miRNA before and after HEN1 knockdown. miRNAs that showed a depletion ≥ two-fold change are indicated in green. The data represent the mean of two independent biological replicates. (B) The relative abundance of miRNA read counts of HEN1 MO vs. control MO are presented in a bar plot. A significant reduction of miRNA read counts is noted in HEN1 MO, (P < .0001, Wilcoxon signed-rank test). The data represents the mean of two independent biological replicates ± SD. (C) The levels of miR-2025, miR-2026, miR-2027 and miR-2028, before and after HEN1 knockdown measured by qPCR using LNA primers. As opposed to miR-2027 and miR-2028, the abundance of mir-2025 and mir-2026 decreased in HEN1 knockdown. The data represents the mean of minimum five independent biological replicates ± SD. ** P ≤ 0.005. * P ≤ 0.05, (Student’s *t*-test). (D) Loss of HEN1 results in shortened *Nematostella* miRNAs. Total reads mapped to guide miRNAs are analysed based on their isoform sizes ranging from 18 to 24 nt. the read counts were presented as the percentage of nucleotide length distribution. In HEN1 MO data the miRNA length is reduced at the percentage of 22 and 23 nt sized isoforms, as indicated with red arrows. (E) miRNAs from HEN1 MO showed accumulation of shorter isoforms. The ratio of percentage of read counts of the most abundant mature miRNA isoform and an isoform shorter by one nucleotide than dominant mature miRNA (“Mature -1nt”) was calculated between the HEN1 MO and control MO the experiment was performed in duplicates and the result is significant at P ≤ 0.01 (Mann-Whitney test). (F) Mean lengths of individual miRNAs were compared between the control MO and HEN1 MO, the data are presented by scatter plot. miRNAs showed in red dots exhibited decrease in mean length in HEN1 MO vs. control MO (P ≤ 0.003, Wilcoxon Signed-Rank Test).

To determine the effect of HEN1 on sRNAs stability in *Nematostella*, we generated and sequenced the sRNA libraries of control and HEN1 MO-injected animals at 3 dpf. The sequenced data was analyzed using miRDeep2 [45] and the raw reads mapped to individual miRNAs were normalized using spike-ins. We compared the total abundance of normalized miRNA read counts in control and HEN1 knockdown animals (Fig 4A; S3 Table). In HEN1 MO-injected animals the levels of ~50% of miRNAs (35 out of 72) dropped more than two-fold (Fig 4A) and overall we observed a significant reduction in miRNA abundance (P < 0.0001, Wilcoxon signed-rank test) (Fig 4B). A similar trend was observed for the translation blocking MO (S2 Fig). To strengthen the results observed from HEN1 knockdown, we carried out periodate treatment on the sRNA collected from the HEN1 and control morphants to test the miRNA stability after HEN1 knockdown. Our analysis revealed that miRNAs of HEN1 morphants were significantly depleted after the treatment (P < 0.0001, Wilcoxon signed-rank test) (S3 Fig), supporting the notion that HEN1 is directly involved in methylation of sRNAs in *Nematostella*.

Notably, the levels of miR-2028 and miR-2027, which were provided as examples for weakly methylated miRNAs (Fig 4A), were not reduced as much as highly methylated miRNAs such as miR-2025 and miR-2026 after HEN1 knockdown (Fig 4A). To provide another support to this observation, the levels of these four miRNAs were validated by quantitative PCR (qPCR), showing that the stability of heavily methylated miRNAs is dramatically reduced in the absence of HEN1 (Fig 4C). The stability of weakly methylated miRNAs is less dependent on HEN1, hence they do not show a dramatic decrease in its absence. It is likely that the increase in miR-2027 and miR-2028 in the experiments (Fig 4C) stems from the diminished relative abundance of other small RNAs, i.e., the depletion of most miRNAs as well as other abundant sRNAs that depend on HEN1-mediated methylation for their stabilization led to miR-2027 and miR-2028 being highly represented in the library.

We hypothesized that the absence of methylation would affect the length of miRNA isoforms as a result of trimming of their 3′ ends by exonucleases. We compared the length distribution of miRNAs in control and HEN1 MO-injected animals. Indeed, we detected a shift towards shorter lengths in HEN1 depleted animals (Fig 4D). The miRNAs of HEN1 morphants showed accumulation of shorter isoforms, when comparing the ratios of miRNA isoforms from control MO vs. HEN1 MO. This is demonstrated by the decrease of the mature isoforms and increase in isoforms which are shorter by 1 nucleotide compared to the mature isoform (“Mature-1nt”) in the morphants (P < 0.01, Mann-Whitney test) (Fig 4E). In addition, when comparing the mean length of the miRNAs depleted by HEN1 knockdown, we detected a significant decrease in the mean length of miRNAs in HEN1 MO injected animals (P < 0.003, Wilcoxon Signed-Rank Test) (Fig 4F). In overall, the analysis suggests that the HEN1 depletion leads to miRNAs lacking 2′-O-methyl at their 3′ end, this subjects the miRNAs to 3′ end trimming, which results in shortening the miRNAs. It is likely that this process leads to the eventual degradation of the miRNAs as reflected in the drop in their total abundance in the morphant animals.

### HEN1 is required for piRNA stability in *Nematostella*

**Fig 5:**
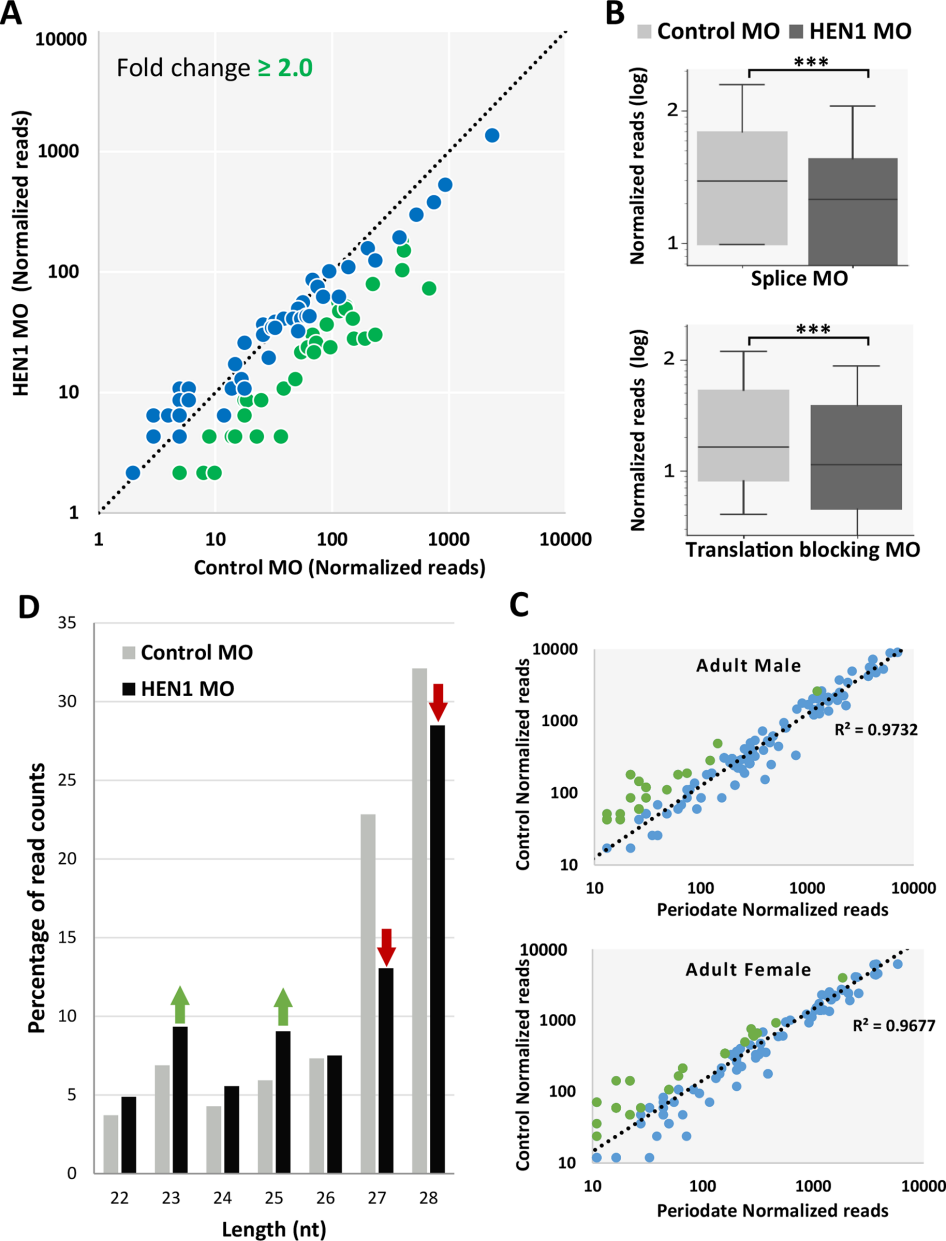
HEN1 is required for piRNA stability. (A) The piRNA read counts were normalized, each dot represents the abundance of an individual piRNA analyzed from HEN1 MO vs. control MO. piRNAs that showed a depletion ≥ two fold are indicated in green. The data represent the mean of two independent biological replicates. (B) The Relative abundance of *Nematostella* piRNAs between HEN1 MO and control MO, in HEN1 MO the piRNA read counts were significantly reduced (P < .0001, Wilcoxon Signed-Rank Test). (C) Scatter plot presenting the change in normalized read counts of individual piRNAs upon periodate treatment. ~90% of piRNAs remained unchanged in abundance upon periodate treatment (indicated in blue). (D) The percentage of nucleotide length distribution plotted for piRNAs reads mapped at 22–28 nt in length. In HEN1 MO data the percentage of 27 and 28 nt sizes was reduced (indicated with red arrows) compared to the control.

In classic bilaterian models such as mammals and nematodes, the HEN1 expression is restricted to the germline [19, 36, 46]. However, the generality of this restriction is now in question due to increasing evidence of somatic piRNAs both in cnidarian and some bilaterian animals [26, 47–50]. In *Nematostella* the expression of the piRNA pathway components is in somatic tissues, and piRNAs are differentially expressed throughout development [51]. We assumed that in addition to miRNAs, methylation and stability of piRNAs are probably also regulated by HEN1 in *Nematostella*. Our sequencing analysis revealed that the levels of about 55% of the tested piRNAs (67 out of 122) exhibited more than two-fold change decrease in HEN1-depleted animals (Fig 5A). Overall, we observed a significant reduction in piRNA abundance (P < 0.0001, Wilcoxon signed-rank test; Fig 5B). This result correlates with our periodate-treatment results, where the majority of piRNAs (~90%) remained stable after the periodate-treatment (Fig 5C). Analysis of periodate treated sRNAs from HEN1 knockdown animals revealed that piRNAs of HEN1 morphants were significantly depleted after the treatment (P < 0.0001, Wilcoxon signed-rank test) (S3 Fig), supporting the notion that HEN1 is directly involved in methylation of piRNAs in *Nematostella*. Next we tested if similarly to bilaterian animals such as zebrafish and *Drosophila*, lack of piRNA methylation would result in trimming of these sRNAs [18, 19, 52]. We plotted the length distribution of piRNAs for both control and HEN1 MO-injected animals. HEN1 depletion shortened the piRNAs (Fig 5D), suggesting that the reduction in piRNAs length likely resulted from trimming of their 3′-ends. Together, our analyses suggest that *Nematostella* HEN1 stabilizes piRNAs by mediating their methylation.

### miRNA and piRNA biogenesis components are essential for *Nematostella* development

**Fig 6:**
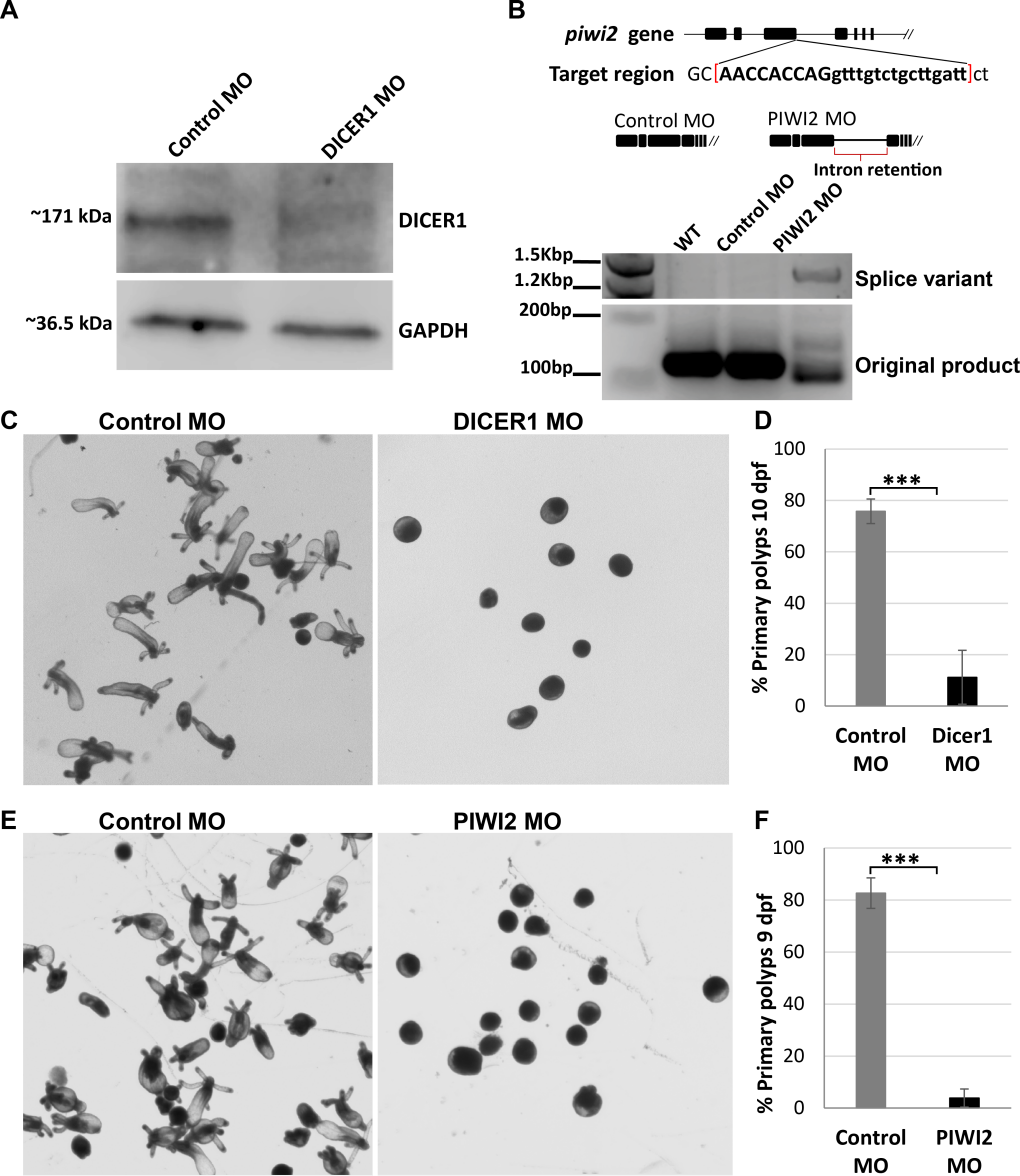
miRNA and piRNA biogenesis components are essential for *Nematostella* development. (A) Western blot analysis using custom *Nematostella* Dicer1 antibody confirms the protein levels were reduced by the knockdown (B) Schematic diagram of MO targeting region on the PIWI2 gene of *Nematostella*. The MO is designed to target PIWI2 exon-intron junction. This MO impaired the splicing by 3^rd^ intron retention of PIWI2 gene. The splicing variation was validated by PCR. Due to intron retention, the PCR product in PIWI2 MO-injected embryos shifted its size. In contrast, the bands in control MO-injected embryos and wildtype presented the expected size. (C) Animals injected with control MO developed to primary polyps after 10 dpf. In contrast, animals injected with Dicer1 MO stopped developing prior to metamorphosis (D) ~76% of Dicer1 depleted animals did not reach primary polyp stage at 10 dpf, experiment performed in triplicates, n = 300, ***P < 0.005 (Student’s *t*-test). (E) Animals injected with control MO developed to primary polyps after 9 dpf. In contrast, animals injected with PIWI2 MO stopped developing prior to metamorphosis (F) ~90% of PIWI2 depleted animals did not reach primary polyp stage at 9 dpf, triplicates, n = 300, ***P < 0.005) (Student’s *t*-test).

In an attempt to discern between the effects of miRNAs and piRNAs defects on *Nematostella* development we knocked down homologs of specific pathway components from Bilateria. Translation blocking MO was used to knockdown Dicer1 (Fig 6A and 6C), a homolog of Dicer, one of the main components in the biogenesis of miRNAs [26, 53]. Western blot analysis using custom Dicer1 antibody confirms the protein levels were reduced by the knockdown (Fig 6A). Splice-inhibiting MO was used to knockdown *Nematostella* PIWI2, a homolog of bilaterian PIWI proteins known to take part in the piRNA ping-pong amplification cycle [8, 23, 51]. *piwi2* splice variants in the MO-injected animals were confirmed by PCR (Fig 6B). Control group of zygotes were injected simultaneously with each treatment, in a similar fashion to the HEN1 knockdowns.

In both Dicer1 and PIWI2 knockdowns most of the MO-injected animals failed to go into metamorphosis and settle as primary polyps (Fig 6C–6F). In the Dicer1 depleted animals, only an average of 11% underwent metamorphosis at 10 dpf, compared to 76% in the control group (Fig 6C and 6D). In the PIWI2 depleted animals only < 10% underwent metamorphosis at 9 dpf, compared to 82% in the control group (Fig 6E and 6F). These results were reproducible in both treatments and were found significant (P < 0.005, Student’s *t*-test). This is a strong indication that both miRNAs and piRNAs are essential for the normal development of *Nematostella*.

**Fig 7:**
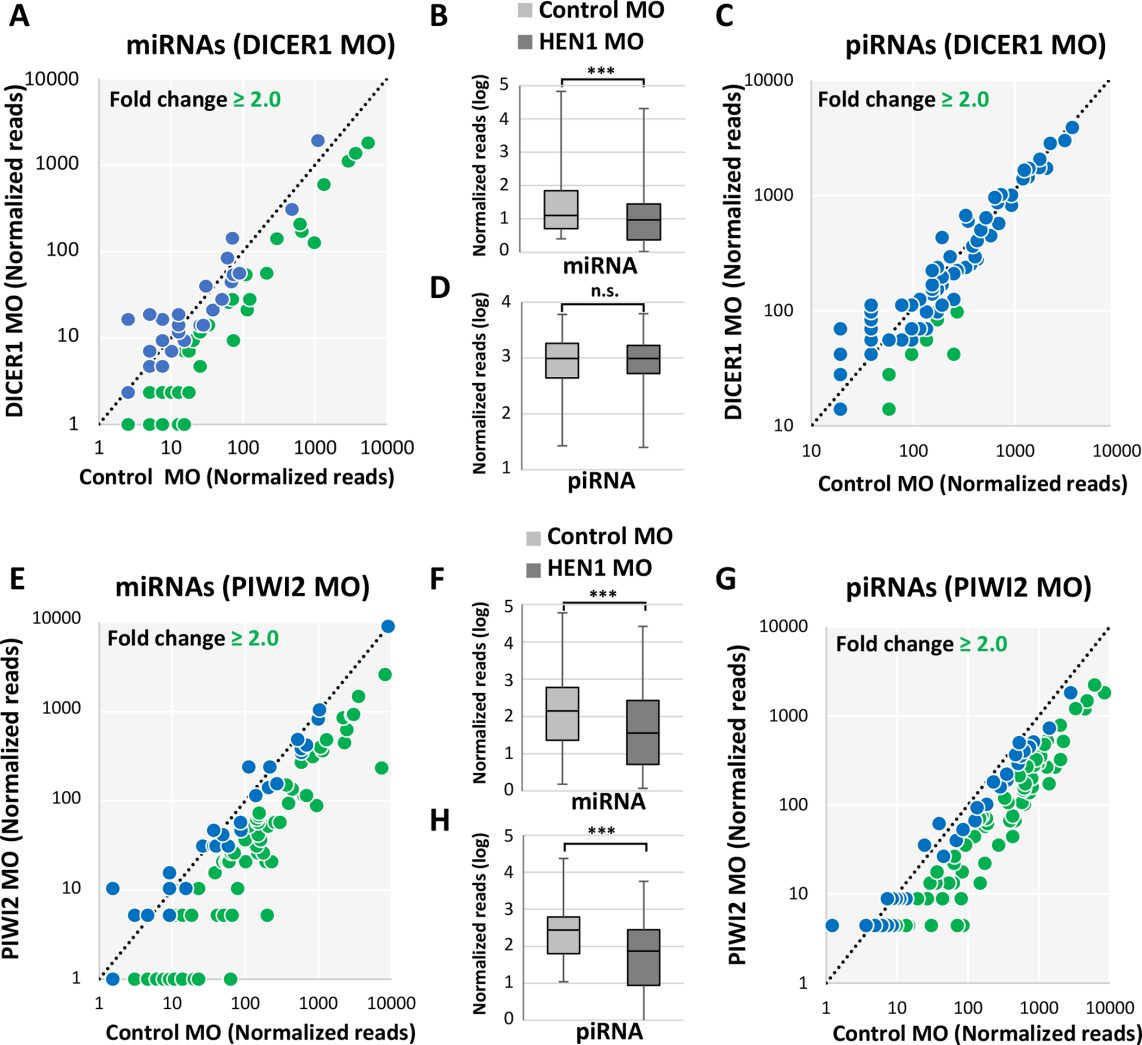
*Nematostella* Dicer1 and PIWI2 knockdown affects sRNA biogenesis. In the scatter plots in panels A, C, E and G the sRNA read counts were normalized, each dot represents the abundance of an individual sRNA analyzed from morphants vs. control. sRNAs that showed a depletion greater than two fold are indicated in green. (A) miRNAs in Dicer1 MO vs. control MO (B) Relative abundance of *Nematostella* miRNAs between Dicer1 MO and control MO, in Dicer1 MO the miRNA read counts were significantly reduced (P < 0.0001, Wilcoxon signed-rank test). (C) piRNAs in Dicer1 MO vs. control MO. (D) Relative abundance of *Nematostella* piRNAs between Dicer1 MO and control MO, in Dicer1 MO the piRNA read counts were not significantly reduced (P = 0.32218, Wilcoxon signed-rank test). (E) miRNAs in Piwi2 MO vs. control. (F) Relative abundance of *Nematostella* miRNAs between Piwi2 MO and control MO, in Piwi2 MO the miRNA read counts were significantly reduced (P < 0.0001, Wilcoxon signed-rank test). (G) piRNAs in Piwi2 MO vs. control MO. (H) Relative abundance of *Nematostella* piRNAs between Piwi2 MO and control MO, in Piwi2 MO the piRNA read counts were significantly reduced (P < 0.0001, Wilcoxon signed-rank test).

To determine the effect of *Nematostella* Dicer1 and PIWI2 knockdown on sRNA populations we sequenced sRNA libraries from Dicer1 and PIWI2 morphants with compatible controls at 2 and 3 dpf, respectively. Data was analyzed similar to HEN1 sRNA libraries. As predicted, in the Dicer1 MO-injected animals a significant drop in the mature miRNAs was observed in comparison to the control MO (P < 0.0001, Wilcoxon signed-rank test) (Fig 7A and 7B) and piRNAs were not affected by Dicer1 knockdown (P = 0.32218, Wilcoxon signed-rank test) (Fig 7C and 7D). Unexpectedly, miRNAs were also affected by the PIWI2 knockdown (P < 0.0001, Wilcoxon signed-rank test) (Fig 7E and 7F). As expected, in PIWI2 MO-injected animals piRNAs were significantly reduced (P < 0.0001, Wilcoxon signed-rank test) (Fig 7G and 7H). In overall, these results suggest that developmental defects observed in *Nematostella* upon HEN1 knockdown are likely contributed by defects in the stabilization of both miRNAs and piRNAs.

## Discussion

In the cnidarian *Nematostella* many miRNAs remain stable after periodate treatment, suggesting that miRNAs are methylated at their 3′-end [22]. In general, methylation of the 3′-terminal nucleotide of miRNAs is part of their biogenesis in plants, but not in animals [34]. Interestingly, in *Drosophila* a handful of miRNAs undergoes 2′-O-methylation and this phenomenon is age-associated [30]. However, we find that in *Nematostella* miRNAs are methylated throughout development (Fig 2B). This is different from the situation in *Drosophila*, where the methylation of a handful of miRNAs is a result of longer isoforms generated with age, being loaded into AGO2 instead of AGO1 [30].

In a recent study we revealed that in *Nematostella*, despite the fact that the majority of miRNAs are expressed as isoforms of various lengths, the dominant guide isoform remains consistent throughout development [28]. Hence, taken together with our current results we suggest that in *Nematostella*, there is a connection between the consistent frequencies of 2′-O-methylation and a stable display of a dominant guide miRNA isoform along development.

**Fig 8:**
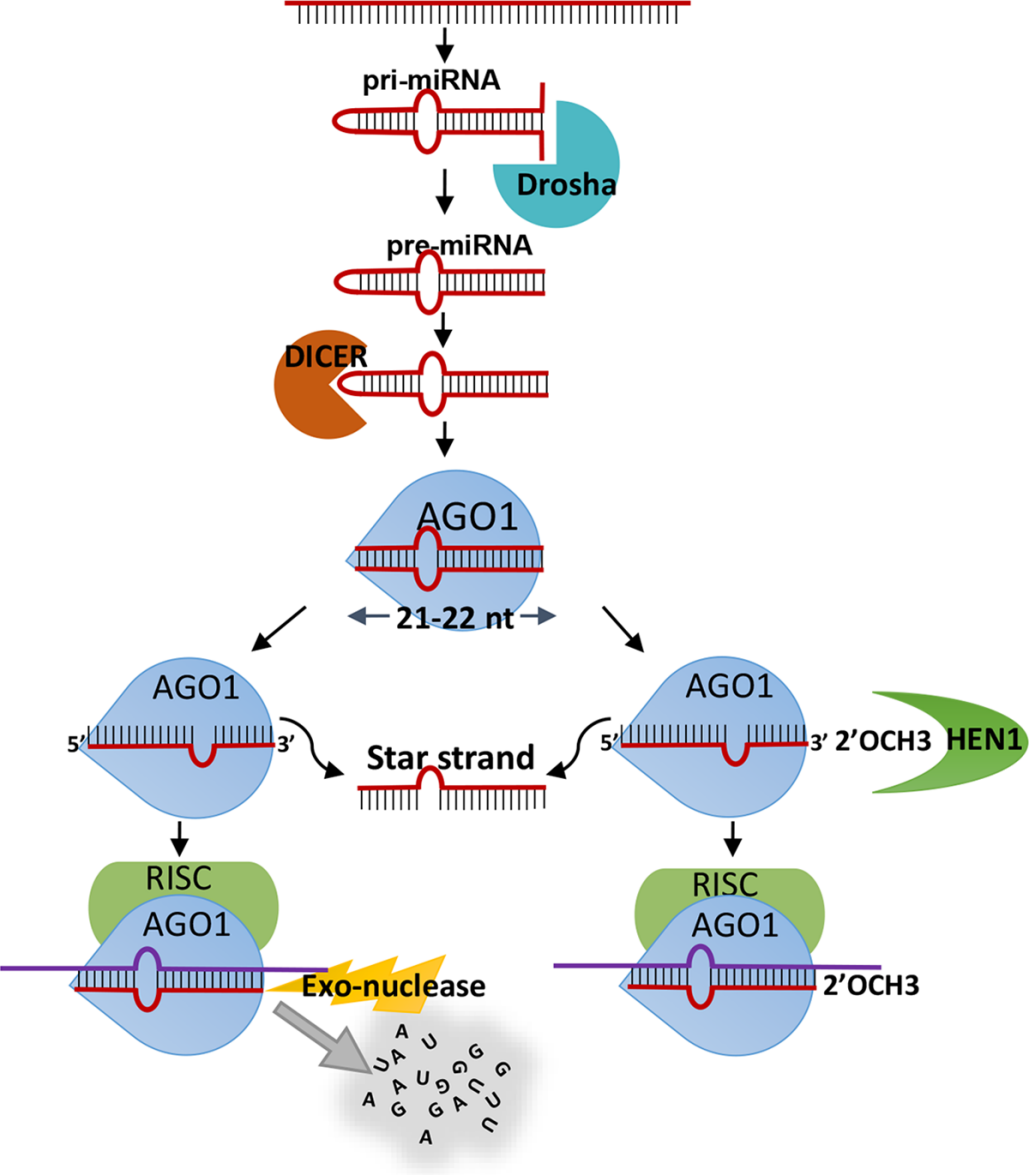
A putative schematic representation of *Nematostella* miRNA biogenesis and methylation of guide miRNA. This scheme is based on the results of the current work as well as results of previous studies in Bilateria [2, 32, 66, 67]. The model suggests that after strand selection by the AGO occurs, the guide strand is methylated by HEN1. When methylation does not occur the guide strand is degraded by exonucleases.

In both plants and bilaterian animals, the 2′-O-methylation at 3′-end of sRNAs is carried out by the methyltransferase HEN1 [33, 34, 54]. HEN1 functional studies carried out in bilaterian animals demonstrated that HEN1 depletion has hardly any effect on miRNA abundance [19]. In a stark contrast, our results in *Nematostella* show that the miRNA abundance significantly dropped in HEN1 depleted animals. We know from degradome sequencing, that *Nematostella* miRNAs interact with their mRNA targets through a high complementary matching throughout their length [22]. Further, based on the proposed “two-state model for Argonaute function”, in case of high complementary matching the 3′-end of miRNA would dissociate from PAZ domain and exposes to 3′-5′exonulceases unlike in the “seed” based target recognition [14, 15, 55] and 3′-end 2′-*O*-methylation is required for protecting the miRNA in such an interaction [21]. These observations in other systems when taken together with our results strongly suggest that in *Nematostella* the HEN1 knockdown has affected the catalysis of 2′-O-methylation at the 3′-end of miRNAs and in turn affected their stability (Fig 4A–4C) and increased the miRNA 3′-end trimming (Fig 4D–4F). In addition, we found that HEN1 in *Nematostella* was able to methylate only the guide strand (miRNA) and not the passenger strand (miRNA*), as can be deduced from the sRNA periodate treatment (Fig 2C and 2D, S1 and S3 Figs). Based on these results, we propose that following the departure of miRNA* from pre-RISC complex, HEN1 interacts with AGO and methylates the guide miRNA strand as a final step in order to stabilize it, as in *Nematostella* guide miRNAs frequently bind their targets via a nearly perfect match (Fig 8).

Similarly to miRNAs, *Nematostella* piRNAs are also significantly decreased due to HEN1 knockdown (Fig 5 and S3 Fig). In bilaterians, unlike miRNAs, the piRNAs are methylated by HEN1 and their stability depends on this modification [18, 19, 41, 56]. As the piRNAs are known to silence the transposable elements of germ cells in metazoans, the knockdown of HEN1 impairs the spermatogenesis and ovulation in bilaterians by modulating piRNA stability [18, 19, 41–44]. In the present study, we observed that HEN1 knockdown inhibited development of *Nematostella* (Fig 3C) and that piRNAs and miRNAs are strongly affected (Fig 4A and 4B) (Fig 5A and 5B).

To determine whether the observed developmental arrest in HEN1 knockdown is due to disruption in miRNAs or piRNAs pathway we knocked down their biogenesis component homologs in *Nematostella*, Dicer1 and PIWI2, respectively. In mice, mutation in the miRNA biogenesis protein Dicer is lethal and double mutants fail to develop [57], while Zebrafish Dicer mutants display brain and heart defects [58]. Loss of the Dicer homolog in plants (DCL1) also results in very early embryonic developmental arrest [59]. All this suggest that developmental role of miRNAs is shared between multiple lineages, yet their function in non-bilaterian animals was never tested. *Nematostella* miRNAs have dynamic and specific spatiotemporal expression patterns and some of their target genes carry developmental roles [22] and hence we speculate that their depletion might have a major contribution to the observed developmental arrest in HEN1 depleted animals. Indeed, we found that Dicer1 knockdown reduces the level of mature miRNAs (but not piRNA) (Fig 7A–7D) and arrests *Nematostella* development (Fig 6C and 6D). These results suggest that the observed phenotype of the HEN1 knockdown might be related to a defect in the miRNA function. This result highlights the potential roles of miRNAs in cnidarian development. However, manipulation of additional biogenesis components will be required in order to substantiate this notion.

Next, we knocked down *Nematostella* PIWI2, hypothesized to take part in piRNA biogenesis [51]. In Bilateria, mutations in *piwi* family genes cause defects in germline development due to their restricted expression to the germline [60–62]. It was found that in the cnidarian *Hydra* piRNAs are present also in somatic cells and have an important role in physiological maintenance [48]. In *Nematostella* piRNA biogenesis components have wide expression domains throughout the body and piRNAs have target sites at transposable elements as well as protein-coding genes and hence might have a role in development [51]. Accordingly, we present that PIWI2 knockdown resulted in a developmental arrest (Fig 6E and 6F) and reduction in the levels of both piRNAs and miRNAs (Fig 7E–7H). The unexpected effect on miRNAs could be non-direct because of changes in wide regulatory networks. Consequently, we could not discern between the roles of the two pathways in this case. These findings support the observations in the HEN1 knockdown and provide a strong indication for the importance of miRNA and piRNA biogenesis and stability to the normal development of *Nematostella*.

We conclude that in the HEN1 morphants, both miRNAs and piRNAs are unmethylated and inherently unstable as indicated by their reduced abundance and length. The loss of HEN1 resulted in developmental abnormalities. To our knowledge, our work is the first functional study of HEN1 in a non-bilaterian animal. Overall, our findings strongly indicate that the last common ancestor of Cnidaria and Bilateria utilized HEN1 to stabilize its piRNAs by 2′-O-methylation that enabled efficient cleavage of transposable elements and possibly other targets. Further, the high complementarity between miRNA and its mRNA targets as well as miRNA 3′ methylation by HEN1 in *Nematostella* is reminiscent of plants and suggest that the common ancestor of plants and animals may have possessed these features. In this scenario, Bilateria, probably due to the acquisition of a seed match based mechanism, have lost the requirement of miRNA 2′-O-methylation by HEN1. While our results provide support for this intriguing evolutionary scenario, it remains to conduct further investigation in order to test its validity.

## Materials and methods

### Animals and microinjection of *Nematostella*

*Nematostella* polyps were grown in 16 ‰ artificial seawater at 18°C in the dark and fed three times a week with freshly hatched *Artemia* nauplii. Induction of spawning was performed as previously described [63]. The gelatinous mass around eggs was removed using 4% L-Cysteine (Sigma-Aldrich, USA) following microinjecting the zygotes with Morpholino antisense oligonucleotide (MO). The zygotes were cultured in 16 ‰ artificial seawater at 22°C in the dark. The MO sequences were designed and synthesized by Gene Tools, LLC (USA). The HEN1 splice MO (5′-3′): AGACTCACAGCCTCACTTACATTTC; HEN1 Translation blocking MO (5′-3′): GTCTCTTTGCGTTTTCATCCCAGAA; Standard control MO (5′-3′): CCTCTTACCTCAGTTACAATTTATA; Dicer1 Translation blocking MO (5′-3′): ATTCCTCTTCGTCACTTGACATCTT; PIWI2 splice MO (5′-3′): AATCAAGCAGACAAACCTGGTGGTT; A 1mM stock solution of each MO was prepared in nuclease-free water. The MO of different treatments and Control MO were injected in equal concentrations on the same day into zygotes from the same batch in order to minimize any genetic or environmental variability. The animals were cultured for 7–10 dpf and the images were collected for morphological analysis. In each independent biological replicate ~300 embryos were microinjected for each MO condition. Samples for RNA or protein extraction were flash frozen in liquid nitrogen and stored at -80°C until used.

### Dicer1 antibody and Western blot

To assess the translation blocking efficiency of Dicer1 MO, western blot with custom antibody was carried out. Custom polyclonal antibodies (Genscript, USA) were generated by recombinant expression of a unique sequence of 153 amino acids within *Nematostella* Dicer1 (positions 487–639 in GenBank Accession AGW15597.1). In each independent biological replicate ~300 embryos were microinjected with Dicer1 MO and control MO. Protein was extracted using RIPA buffer (1M Tris pH 7.4, 0.1M Dithiothreitol, 1.5M KCl, 0.5M EDTA, 10% NP-40) with protease inhibitors (Roche cOmplete ULTRA tablets and Merck proteas inhibitor cocktail set III). The PVDF membranes (BioRad, USA) carrying the protein extract after blotting were blocked at room temperature for 1 h in blocking solution containing 5% dry milk in TBST (Tris-buffered saline and 0.1% Tween). Each membrane was divided at ~90 kDa and parts were incubated with primary antibodies in a solution containing 5% Bovine serum albumin (BSA) in TBST: Custom Guinea pig anti-Dicer1 primary antibody at 1μg/ml and Rabbit anti-GAPDH antibody diluted 1:1,000 (Catalog no. ab9485-100, Abcam) for normalization. Membranes were then incubated over night at 4°C. After three washes with TBST for 10 min each, membranes were incubated for 1 h at room temperature in Donkey-anti-Guinea pig (Catalog no. 706-035-148, Jackson immunoresearch) or Goat anti-Rabbit (Catalog no. 111-035-144, Jackson immunoresearch) HRP-conjugated secondary antibodies diluted 1:10,000 in a solution containing 5% dry milk in TBST. Membranes were developed with Clarity ECL substrate (BioRad). This experiment was carried out at three biological replicates.

### Periodate treatment

Total RNA was extracted from different developmental stages of *Nematostella* (late planula, primary polyp, adult male and female) using Tri-Reagent (Sigma-Aldrich) following the manufacturer′s instructions. Three biologically independent animal pools were used for each developmental stage. From each stage, 20 μg of total RNA was used and the RNA integrity was analyzed with a Bioanalyzer (Agilent Technologies, USA). The small RNA size selection was performed using 15% denaturing urea polyacrylamide gel (BioRad), RNA elution from the gel was performed overnight and precipitated using ethanol. The sRNA was divided into equal portions, one portion was subjected to periodate treatment and another portion served as a control. The periodate treatment was executed at 25°C for 30 min, using 50 mM sodium periodate (Sigma-Aldrich) in 5× Borate buffer (pH 8.6). The sRNA was precipitated using ethanol and used as input for sRNA library preparation. Similar periodate treatment was implemented on sRNAs collected from HEN1 and control morphants to assess the HEN1 depended sRNA methylation.

### Small RNA library preparation

The animals injected with HEN1 and PIWI2 MO were collected at 3 dpf and Dicer1 were collected at 2 dpf. Total RNA extraction and size selection were carried out as explained above. Two biologically independent animal pools (~150 larvae) were collected from each MO condition together with it compatible control. Prior to sRNA library preparation four synthetic bilaterian miRNA were added as spike-ins to the total RNA, among them two were 2′-O-methylated at their 3′ end (mmu-miR-125a-5p and mmu-miR-148a-3p) and two were non-methylated (cel-lin-4-5p and hsa-miR-659-5p). sRNA libraries were prepared as described in the Zamore Lab Illumina TruSeq small RNA Cloning Protocol April 2014 (http://www.umassmed.edu/zamore/resources/protocols/). In brief, sRNAs were ligated to 3′ and 5′ adapters containing 4 random nucleotides at the ligation interface to minimize ligation bias. Ligation products were reverse transcribed using SuperScript III Reverse Transcriptase (Thermo Fisher, USA) and cDNA samples were PCR amplified using KAPA Real-Time Library Amplification Kit (PeqLab, Germany). Amplified cDNA was purified on 2% agarose gels, followed by sRNA library sequencing on NextSeq 500 (Illumina, USA) with 50 nt read length. The raw data have been deposited at NCBI GEO Submission (GSE94526) (GSE110148) and (GSE115184) (S1 Table).

### Bioinformatic analysis

The sequencing data were pre-processed to remove the adapters using Cutadapt [64] and sequences shorter than 18 nt were discarded. Processed data were analyzed using miRDeep2 core algorithm to identify any new miRNAs and also to assess the authentic mature miRNA sequences [45]. The *Nematostella* genome from the NCBI database was used as a reference, and the mature miRNA and miRNA precursor were retrieved from miRBase Release 21 [65]. Among the mature miRNA reads mapped to miRNA precursors, based on the read counts we considered the sequence with the higher read count as the guide strand and the less abundant opposite strand as the star strand. For miRNA quantification, the miRDeep2 quantification algorithm was used. In order to determine the methylated miRNAs through quantification, we used spike-ins for normalizing the read counts. The read counts of the methylated spike-in mmu-miR-148a-3p was used for normalization of sRNA reads in experiments that included periodate treatment. For the rest of experiments, sRNA read counts were normalized to the average of all four spike-ins. The normalized read counts among treated and control samples was analyzed and drawn as a scatter plot. To determine the methylation levels during development we compared the log_2_ fold change among all different time points and plotted on heat map using MultiExperiment Viewer (MeV version 4.7) (http://mev.tm4.org/). The nucleotide size distribution was analyzed from the mapper file generated using miRDeep2 in arf format, with read counts mapped to the reference *Nematostella* miRNA sequence list retrieved from miRBase Release 21. For piRNA analysis, the 3′ adapter was clipped using cutadapt and pre-processed sequence data was mapped to a list of 171 piRNAs previously identified from *Nematostella* genome [51]. The read length distribution analysis was carried out on piRNA reads mapped between 22 to 28 nt length.

### Reverse transcription and quantitative real-time PCR (qPCR)

Reverse transcription (RT) reaction for miRNA assay was performed using miRCURY LNA Universal RT microRNA PCR Kit (Catalog no. 339340; Exiqon-Qiagen, Denmark), the RT was carried out as instructed in miRCURY LNA RT Kit manual, an equal amount of RNA spike-in (Uni-Sp6) was added into RNA and later used as internal extraction and amplification control. The RT mixture included the template RNA (150 ng), 5x miRCURY RT Reaction Buffer (2 μl), 10x miRCURY RT Enzyme Mix (1 μl) and nuclease-free water up to 10 μl total volume. The mixture was Incubate for 60 min at 42°C, then incubate for 5 min at 95°C to heat inactivate the reverse transcriptase and immediately cool to 4°C. Real-Time PCR assay was performed using miRCURY SYBR Green PCR Kit (Catalog no. 339345; Exiqon-Qiagen) and the Reactions were performed according to the manufacturer’s instructions using StepOnePlus Real-Time PCR System (ABI instrument, Thermo Fisher). The qPCR mixture includes 2x miRCURY SYBR Green Master Mix (5 μl), LNA primer set (1 μl), cDNA template (3 μl) and nuclease-free water up to 10 μl total volume. qPCR thermocycling conditions were as follows: 95°C for 2 min, followed by 40 cycles of 95°C for 10 s, 56°C for 1 min, melt curve analysis were performed between 60–95°C for 15 min at a ramp-rate of 1.6°C/s. Minimum five independent biological replicates were used for each HEN1 MO condition and all samples were run as technical triplicates. The expression levels of target miRNAs were normalized to the RNA spike-in (Uni-Sp6).

## Supporting information

S1 Fig In *Nematostella vectensis* miRNA* are unmethylated. (A) A box plot presenting the Log-fold change of four individual spike-ins analyzed between samples treated with periodate and control samples. The two non-methylated spike-ins (Cel-lin-4 and has-miR-659) significantly changed after periodate treatment (~1000 fold change). (B) A list of highly abundant miRNAs were selected and depicted their log_2_ fold change on the heatmap. (C) A scatter plot presenting the mean of log_2_ fold change upon periodate treatment for all miRNAs from different developmental stages (late planula, primary polyp, adult female and adult male). The greater portion of miRNA* (blue dots) have higher fold change when compare to miRNA guide (green dots).
(TIF)

S2 Fig HEN1 translation blocking Morpholino reproduce similar results to HEN1 splice Morpholino on both *Nematostella* development and small RNA stability. (A) Animals injected with HEN1 translation blocking Morpholino have stopped developing prior to metamorphosis. (B) ~90% of HEN1 depleted animals did not reach primary polyp stage at 7 dpf, n = 3, significant at P < 0.001 (Student’s *t*-test). (C-D) HEN1-TB MO effected the stability of both miRNA and piRNA population. Data represented as mean of two independent biological replicates.
(TIF)

S3 Fig HEN1 directly mediates miRNA and piRNA methylation in *Nematostella*. Box plot presented with log fold change analyzed from periodate vs untreated data of HEN1 and control morphants. (A) The miR-Guide fold change analyzed from HEN1 morphants and control MO animals after periodate treatment has significantly changed (P < .0001, Wilcoxon signed-rank test) in HEN1 morphants. (B) The miR-Star (passenger strand) fold-change analyzed from HEN1 morphants and control animals from periodate treatment remain insignificant (P = 0.15854, Wilcoxon signed-rank test). (C) The piRNAs fold change analyzed from HEN1 and control Morphants after periodate treatment has significantly changed (P < .0001, Wilcoxon signed-rank test) in HEN1 morphants.
(TIF)

S1 Table Small RNA libraries produced in this study. (XLSX)

S2 Table List of miRNAs analyzed from different developmental stages of *Nematostella* (late planula, primary polyp, adult male and female), to assess the rate of miRNA methylation across the development. (XLSX)

S3 Table List of miRNAs and piRNAs analysed from control MO and HEN1 MO-injected animals. (XLSX)

S4 Table List of miRNAs and piRNAs analysed from Dicer1 and PIWI2 knockdown experiments. (XLSX)
