# Supplementary figures and images for: The methyltransferase HEN1 is required in Nematostella vectensis for microRNA and piRNA stability as well as larval metamorphosis
Source: PLoS Genet. 2018 Aug 17;14(8):e1007590. doi: 10.1371/journal.pgen.1007590 (PMC6114907; doi:10.1371/journal.pgen.1007590)

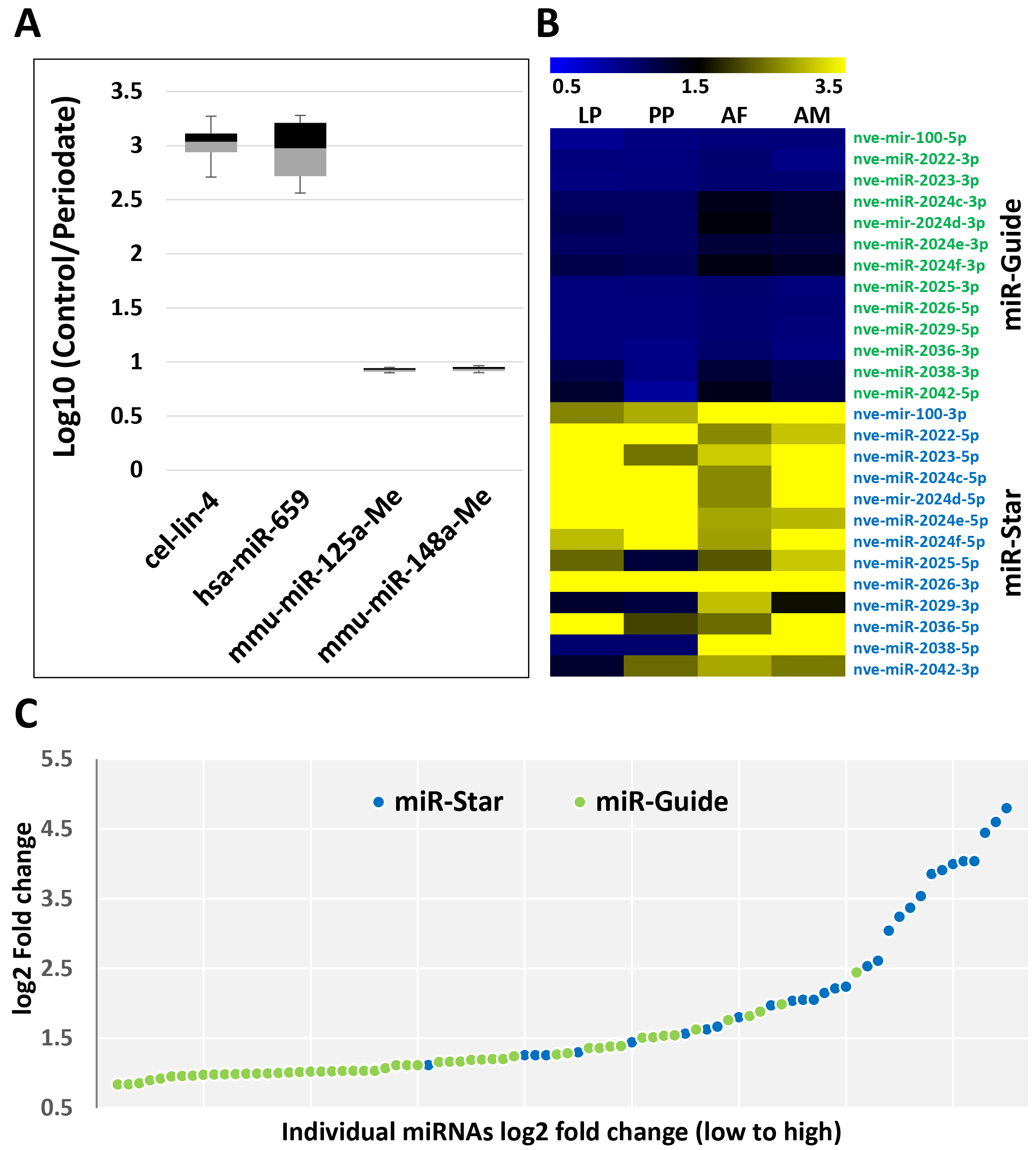

Supplement: S1 Fig — (A) A box plot presenting the Log-fold change of four individual spike-ins analyzed between samples treated with periodate and control samples. The two non-methylated spike-ins (Cel-lin-4 and has-miR-659) significantly changed after periodate treatment (~1000 fold change). (B) A list of highly abundant miRNAs were selected and depicted their log2 fold change on the heatmap. (C) A scatter plot presenting the mean of log2 fold change upon periodate treatment for all miRNAs from different developmental stages (late planula, primary polyp, adult female and adult male). The greater portion of miRNA* (blue dots) have higher fold change when compare to miRNA guide (green dots). (TIF) [file pgen.1007590.s001.tif]

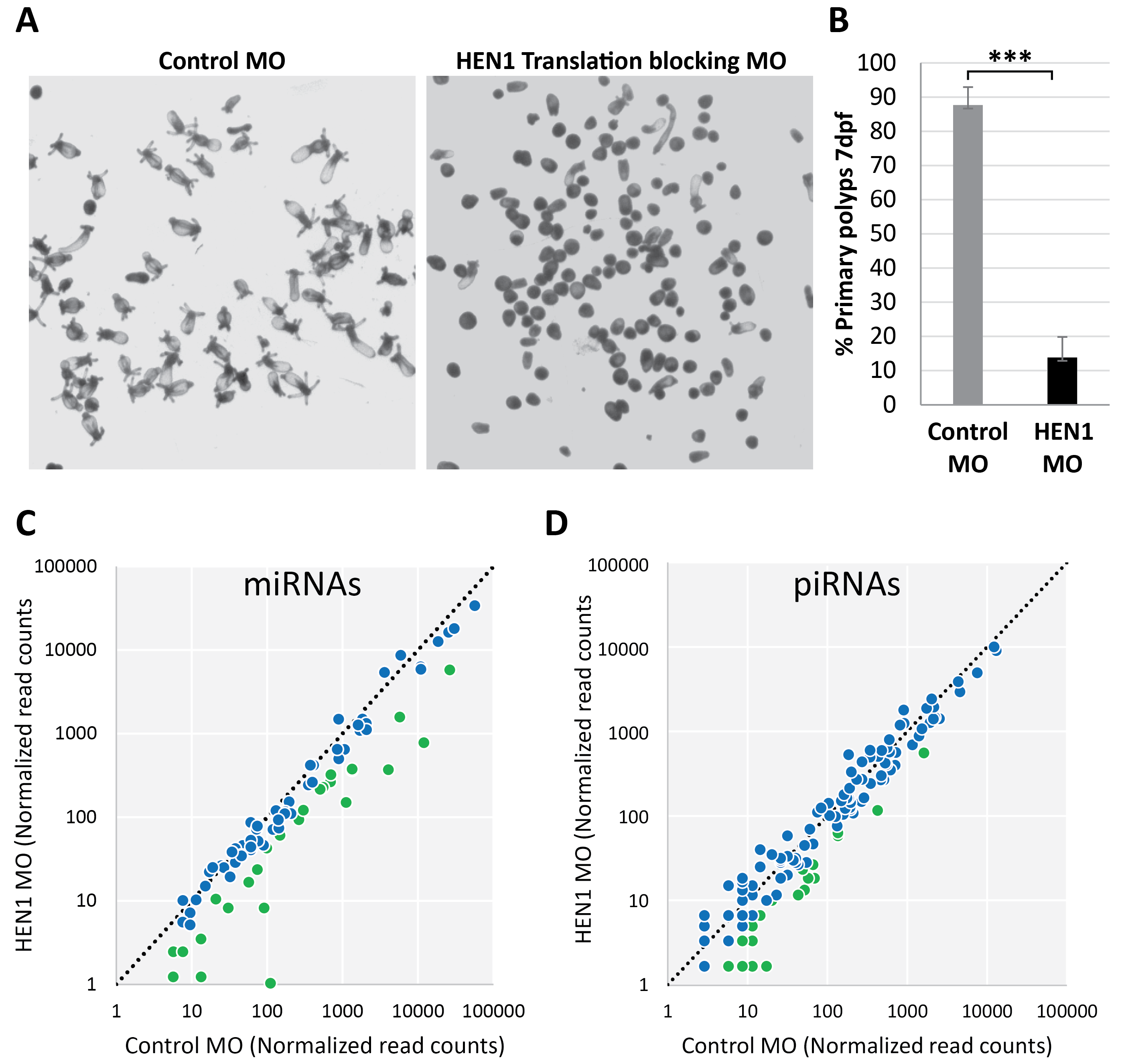

Supplement: S2 Fig — (A) Animals injected with HEN1 translation blocking Morpholino have stopped developing prior to metamorphosis. (B) ~90% of HEN1 depleted animals did not reach primary polyp stage at 7 dpf, n = 3, significant at P < 0.001 (Student’s t-test). (C-D) HEN1-TB MO effected the stability of both miRNA and piRNA population. Data represented as mean of two independent biological replicates. (TIF) [file pgen.1007590.s002.tif]

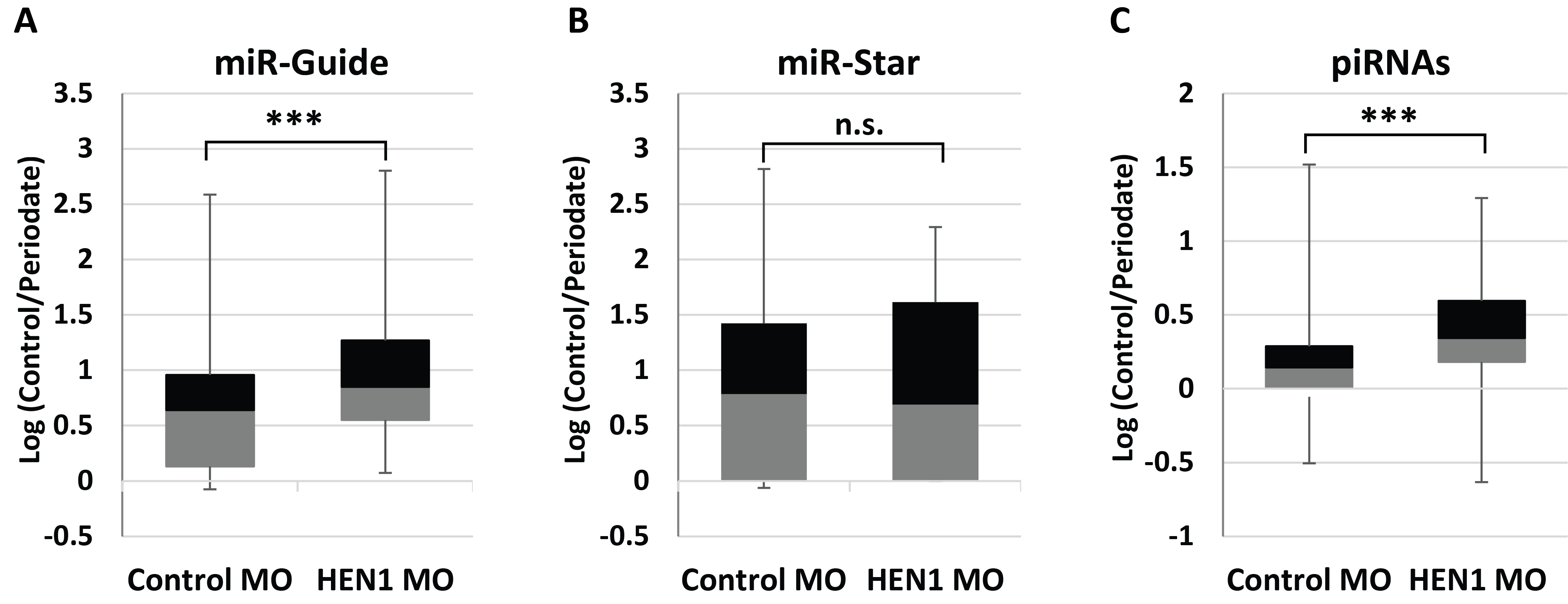

Supplement: S3 Fig — Box plot presented with log fold change analyzed from periodate vs untreated data of HEN1 and control morphants. (A) The miR-Guide fold change analyzed from HEN1 morphants and control MO animals after periodate treatment has significantly changed (P < .0001, Wilcoxon signed-rank test) in HEN1 morphants. (B) The miR-Star (passenger strand) fold-change analyzed from HEN1 morphants and control animals from periodate treatment remain insignificant (P = 0.15854, Wilcoxon signed-rank test). (C) The piRNAs fold change analyzed from HEN1 and control Morphants after periodate treatment has significantly changed (P < .0001, Wilcoxon signed-rank test) in HEN1 morphants. (TIF) [file pgen.1007590.s003.tif]
